# Fertilizing a Patient Engagement Ecosystem to Innovate Healthcare: *Toward the First Italian Consensus Conference on Patient Engagement*

**DOI:** 10.3389/fpsyg.2017.00812

**Published:** 2017-06-06

**Authors:** Guendalina Graffigna, Serena Barello, Giuseppe Riva, Mariarosaria Savarese, Julia Menichetti, Gianluca Castelnuovo, Massimo Corbo, Alessandra Tzannis, Antonio Aglione, Donato Bettega, Anna Bertoni, Sarah Bigi, Daniela Bruttomesso, Claudia Carzaniga, Laura Del Campo, Silvia Donato, Silvia Gilardi, Chiara Guglielmetti, Michele Gulizia, Mara Lastretti, Valeria Mastrilli, Antonino Mazzone, Giovanni Muttillo, Silvia Ostuzzi, Gianluca Perseghin, Natalia Piana, Giuliana Pitacco, Gianluca Polvani, Massimo Pozzi, Livio Provenzi, Giulia Quaglini, Mariagrazia Rossi, Paola Varese, Natalia Visalli, Elena Vegni, Walter Ricciardi, A. Claudio Bosio

**Affiliations:** ^1^Faculty of Psychology, Università Cattolica del Sacro CuoreMilan, Italy; ^2^Istituto Auxologico Italiano (IRCCS)Milan, Italy; ^3^Istituto Nazionale dei Tumori (IRCCS)Milan, Italy; ^4^Casa di Cura Privata del PoliclinicoMilan, Italy; ^5^Faculty of Economics, Università Cattolica del Sacro CuoreMilan, Italy; ^6^Federazione Italiana delle Associazioni di Volontariato in Oncologia (FAVO)Rome, Italy; ^7^Fatebenefratelli Ospedale Sacra FamigliaComo, Italy; ^8^Department of Linguistics, Università Cattolica del Sacro CuoreMilan, Italy; ^9^Società Italiana di Diabetologia (SID)Rome, Italy; ^10^Dipartimento di Medicina, Azienda Ospedaliero-Universitaria di PadovaPadova, Italy; ^11^Gruppo Italiano Infermieri di Area Cardiovascolare (GITIC)Rome, Italy; ^12^Dipartimento di Scienze Sociali e Politiche, Università degli Studi di MilanoMilan, Italy; ^13^Department of Economics, Management and Quantitative Methods, Università degli Studi di MilanoMilan, Italy; ^14^Associazione Nazionale Medici Cardiologi Ospedalieri (ANMCO)Rome, Italy; ^15^Ordine degli Psicologi del LazioRome, Italy; ^16^Italian Ministry of Health, Prevention General DirectionRome, Italy; ^17^Federazione delle Associazioni dei Dirigenti Ospedalieri Internisti (FADOI)Rome, Italy; ^18^Collegio IPASVI Milano – Lodi – Monza e BrianzaMilan, Italy; ^19^Associazione Lombarda Malati Reumatici (ALOMAR)Milan, Italy; ^20^Interdepartmental University Research Center on Motor Activity, University of PerugiaPerugia, Italy; ^21^Azienda Sanitaria Universitaria Integrata di TriesteTrieste, Italy; ^22^Centro Cardiologico Monzino (IRCCS)Milan, Italy; ^23^Dipartimento di Scienze Cliniche e di Comunità, Università degli Studi di MilanoMilan, Italy; ^24^0-3 Center for the at-Risk Infant, IRCCS Eugenio MedeaBosisio Parini, Italy; ^25^Confederazione Parkinson Italia ONLUSMilan, Italy; ^26^Associazione Medici Diabetologi (AMD)Rome, Italy; ^27^Department of Health Sciences, Università degli Studi di MilanoMilan, Italy; ^28^Istituto Superiore di SanitàMilan, Italy

**Keywords:** patient engagement, consensus conference, Italy, chronic care

## Abstract

Currently we observe a gap between theory and practices of patient engagement. If both scholars and health practitioners do agree on the urgency to realize patient engagement, no shared guidelines exist so far to orient clinical practice. Despite a supportive policy context, progress to achieve greater patient engagement is patchy and slow and often concentrated at the level of policy regulation without dialoguing with practitioners from the clinical field as well as patients and families. Though individual clinicians, care teams and health organizations may be interested and deeply committed to engage patients and family members in the medical course, they may lack clarity about how to achieve this goal. This contributes to a wide “system” inertia—really difficult to be overcome—and put at risk any form of innovation in this filed. As a result, patient engagement risk today to be a buzz words, rather than a real guidance for practice. To make the field clearer, we promoted an Italian Consensus Conference on Patient Engagement (ICCPE) in order to set the ground for drafting recommendations for the provision of effective patient engagement interventions. The ICCPE will conclude in June 2017. This document reports on the preliminary phases of this process. In the paper, we advise the importance of “fertilizing a patient engagement ecosystem”: an oversimplifying approach to patient engagement promotion appears the result of a common illusion. Patient “disengagement” is a symptom that needs a more holistic and complex approach to solve its underlined causes. Preliminary principles to promote a patient engagement ecosystem are provided in the paper.

The debate on patient engagement has dramatically increased in the past 10 years with a peak in scientific publications in 2015. Scholars agree on the urgency of engaging patients in their care in order to achieve a more sustainable management of the healthcare system (Fisher et al., 2016; O’Hara and Lawton, 2016; Weil, 2016). Low-resource healthcare systems face increased organizational healthcare costs, which is likely to result in an allocation of limited health resources (Tinetti and Basch, 2013; Hussey et al., 2014). All governments and societies face rising demand for services and higher quality of life expectations, and they need to reconcile these requests with the limited resources availability (Parekh et al., 2011; Edelman et al., 2013). Engaging patients may play a crucial role in the co-design of care services, in order to improve its clinical efficacy and its organizational efficiency (Richards et al., 2015; Gilardi et al., 2016). Notably, the US Affordable Care Act also endorses patient engagement because without it, even with best practices on the part of healthcare providers, it is very difficult to achieve optimal health outcomes and constrain costs (Kennedy et al., 2007; Sommers and Bindman, 2012). This is particularly crucial for the Italian Healthcare System, due to its tax-funded nature (Fattore and Torbica, 2005): in the recent period of economic crisis in Italy, policy makers as well as healthcare professionals are struggling to find innovative solutions to improve the efficacy and efficiency of the their system (de Belvis et al., 2012).

Whilst the “whys” for opting for the patient engagement solution are clear enough, there is still no consensus on how to achieve this goal. *Is there a magic potion for patient engagement? What are the key recommendations for achieving the patient engagement goal?*

To overcome this challenge, the Italian Consensus Conference on Patient Engagement (ICCPE) aimed at developing greater partnership among all the healthcare stakeholders (policy makers, clinicians, patient, and family representatives) by working on sharing and disseminating research evidence and good practices from the field.

## Methods

The ICCPE started in November 2015 and is going to be concluded in June 2017, according to the standard of the Consensus Development Programs of the American National Institutes of Health (NIH). The panel included 104 experts belonging to different disciplinary backgrounds (i.e., medicine, psychology, sociology, education, rehabilitation, nursing, management, public health, policy making, health engineering) in order to promote a trans-disciplinary debate and to reach a broader consensus. The ICCPE also included representative from patients, families, and voluntary associations on behalf of both their community and their direct illness experience. The ICCPE process included the following steps. (1) Workshops with the scientific board to first achieve a shared definition of the objectives and protocol of the ICCPE, to list a set of questions to be addressed and to orient expert group works. (2) Therefore, a systematic literature review was conducted on the main scientific databases (i.e., PUBMED, SCOPUS, COCHRANE, and ISI WEB OF SCIENCE) aiming at detecting all the initiatives, programs, strategies, and tools intended to improve patient engagement. (3) In the following phase, oral and written feedback on the feasibility and priority of the strategies detected from the literature was collected from the experts. Furthermore, experts were invited to give oral presentation of their best practices for promoting patient engagement. (4) Then, experts were requested to fill in a questionnaire to systematically evaluate and grade the strategies for promoting patient engagement which they considered the most effective. (5) In a last workshop, a document integrating the evidences from the systematic analysis of the literature and all the experts’ feedback was deeply discussed for revision, integration, and approval.

**FIGURE 1:**
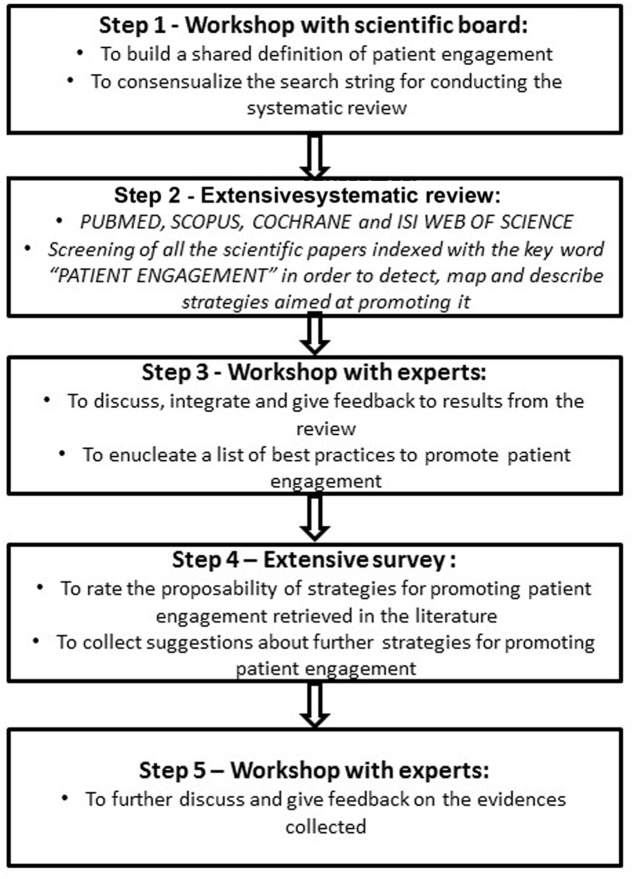
**The preliminary phases of the ICCPE**.

In the following paragraphs, we describe some preliminary insights emerged from these early phases of the ICCPE (see **Figure 1**).

## “The Diagnosis”: *Mind the Following Challenges to Patient Engagement*

### First: *Fragmentation and Oversimplification May Lead to Failure*

The systematic search of the literature produced 2846 records indexed with the key expression “patient engagement.” However, out of the 2846 records retrieved, only 104 described real actions and tools for patient engagement, testifying to gaps in the translation of theories into practices. Still prevalent among the patient engagement interventions retrieved in the literature was a simplified and fragmented approach to the individual actors involved in the care process. The vast majority of studies only addressed individual patients as the main target of the interventions (56% of the retrieved sources), thus testifying to the implicit (simplistic) assumption that to achieve engagement the only actor that needs to be changed is the patient. Few initiatives were dedicated to other actors (such as training and support for clinicians, 18%; educational initiatives targeted on family and caregivers, 5%). Furthermore, only a minority of sources (19%) featured synergic interventions addressing different actors and at different levels of the healthcare organization (i.e., micro, meso, macro). This fragmented approach appears to experts inadequate, given the complexity of the patient engagement phenomenon and the multiple factors involved in its promotion. Experts also consider this issue like symptomatic of a “medicalizing” tendency: the majority of interventions retrieved in the literature (but also reported by the experts in the workshops) aimed at “correcting” patients’ attitudes and behaviors in order to make them better able to self-manage (and more adherent to medical prescriptions). By contrast, very few contributions retrieved along the process aimed at critically revising the overall process of services delivery in order to enhance co-production and the joint participation of the various actors involved (i.e., patients, caregivers, clinicians, but also healthcare managers, society, patients’ associations, institutions, and so forth) (Sabadosa and Batalden, 2014).

### Second: *One Size Does Not Fit All*

Experts pointed at the fact that patient engagement interventions are frequently standardized and “fixed *a priori*” without deep and actual personalization. Rarely they are interventions based on the profiling of patients according to their clinical, socio-cultural and psychological characteristics. Moreover, the practice of assessing the level of patient engagement is still far from being a routine in several healthcare settings. Patient engagement is often considered like an “on–off” condition, forgetting that it is a psycho-social and dynamic, fluid and mutable experience. Experts suggest that closer attention to patients’ subjectivity and to how patient engagement changes and evolves along the care pathway may orient more applicable and personalized interventions. Moreover, assessment of the subjective experience of the other actors involved in the care process, of their needs for support and their expectations—neglected in the majority of cases—may furnish important insights and new perspectives for the promotion of patient engagement.

### Third: *Disengaged Clinicians Are a Hindrance to Effective Patient Engagement Initiatives*

Experts pointed at the fact that the patient engagement imperative is often a top-down prescription for clinicians. Doctors may experience it as a further professional duty to be performed and find it irksome. Furthermore, the organizational change toward patient engagement may deteriorate clinicians’ organizational commitment and work engagement. There ensue two negative consequences: decreased clinicians’ wellbeing and lower compliance with patient engagement initiatives. Clinicians also complain that they lack the knowledge and skills needed to successfully achieve the goal of patient engagement in their clinical practice: this, according to experts, may cause frustration and burnout.

### Fourth: *The Patients’ Family Caregivers Deserve to Be Engaged As Well*

More and more patients are not alone when navigating the healthcare system. The family—emotionally and practically—sustains patients in their healthcare process; this happens particularly in the Italian healthcare system due to cultural and pragmatic issues. This is particularly evident in the case of high-intensity care conditions or disabilities, where patients cannot autonomously manage their care. However, caregivers are an under-estimated source of patient engagement. However, too often, from experts, the “engagement problem” is circumscribed to individual patients, losing sight of the needs, preferences and expectations of their family caregivers. Interventions aimed at fostering family caregiver engagement are today few. From experts’ perspective, the lack of consideration of the family caregivers’ role in the patient engagement process is not only missed opportunity to enhance the effectiveness of intervention but also a major risk to the success of ongoing initiatives to promote patient engagement targeted on individual patients.

## ”The Therapy”: Principles for Promoting Effective Patient Engagement

### First: *Fertilize a Patient Engagement Ecosystem!*

**FIGURE 2:**
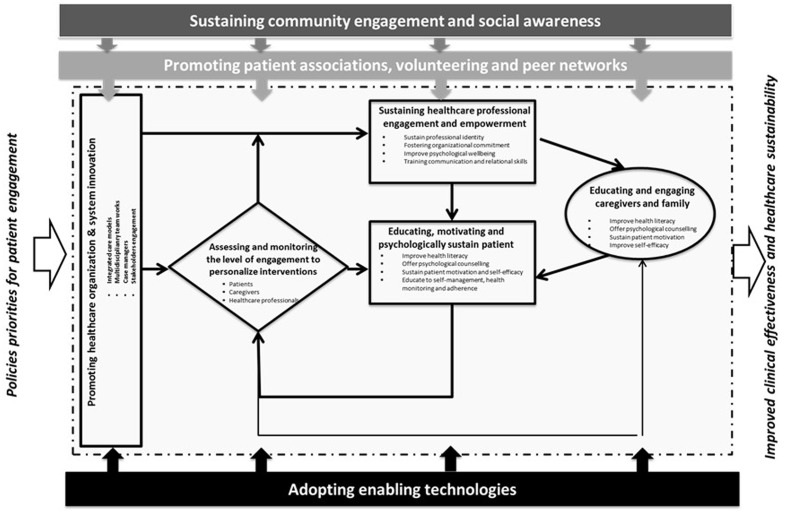
**The patient engagement eco-system**.

As argued above, an oversimplifying approach to patient engagement promotion appears to result from a widespread illusion. Patient “disengagement” is a symptom that requires a more holistic and complex approach to solving its underlying causes. According to experts, seeking the “magic potion” to make individual patients engaged may not be the right answer: rather, a systemic and multilevel approach to patient engagement, involving all the different actors in pursuit of a common goal, may be the solution to maximize benefits. Patient engagement is a new paradigm in healthcare service organization and delivery. Its purpose is to obtain the cooperation and the “co-authorship” of all the different actors. This means fertilizing and fostering an organizational environment able to sustain patient engagement. In particular this implies the planning of synergic actions to promote patient engagement addressed to the multi-layered factors affecting this process (“patient engagement ecosystem”). Society, for instance, should also be a target for patient engagement initiatives: communities need to be sensitized, educated and supported to best sustain patients and families in their engagement. Patients’ associations and voluntary networks are crucial assets with which to promote patient and family advocacy initiatives and must be taken on board. Finally, new technologies may enable the patient engagement ecosystem: they should be envisaged not as the ultimate goal of patient engagement initiatives, but rather as important supports for systemic synergy among the actors and the stakeholders involved in the process (**Figure 2**).

### Second*: Make Patient Engagement Measurement a Routine!*

According to experts, a first crucial step in fostering a patient engagement ecosystem is to implement routine processes of measuring patient (and their caregivers) engagement. Validated measures of patient engagement may fulfill several purposes. First, they may constitute a powerful communication and advocacy tool to “give voice” to patients and their families about their care experiences, their needs and their preferences. This may enable healthcare organizations and clinicians to align their initiatives more closely with the specific preferences of care receivers and to identify those more at risk of disengagement: not always does the expert perspective perfectly mirror that of patients and caregivers. Furthermore, patients’ needs change and evolve along the care pathway, and they must be constantly assessed and considered: this is the only way to ensure personalization of intervention and the incorporation of patients and family caregivers’ perspectives in the design and delivery of healthcare services. Finally, experts advocate for healthcare organizations testing the effectiveness of their initiatives and optimizing them to enhance clinical effectiveness and economic sustainability by adopting reliable measures of patient engagement.

The experts, however, suggest a broader practice which assesses also the work engagement levels and needs of clinicians, and which measures the level of engagement of caregiver families. Only a multi-stakeholder approach to engagement measurement can realistically offer insights for the realization of a patient engagement ecosystem. Thus, not only should already validated measures of patient engagement be adopted but new ones specifically dedicated to assessing the engagement experiences and needs of all the actors involved in the process should be developed.

### Third: *Clinicians Must Be Engaged Too!*

Experts believe clinicians need to be supported and equipped so that they can best accomplish the patient engagement goal. They need to foster their work engagement and organizational commitment as an antidote to the risk of burnout, and as a crucial asset for improving patient engagement. To achieve this goal, healthcare organizations must first sensitize clinicians to the clinical and organizational benefits of opting for patient engagement: the sharing and discussion of scientific literature, seminars, workshops, conferences, continuing and distance education are fundamental tools with which to make patient engagement become a shared goal of clinicians, rather than being a prescription to comply with. Furthermore, in order to become truly able to engage patients, clinicians need to be sustained in revising their professional identity in light of the empowered patient: burnout assessment, work engagement interventions and psychological consultancy are some important practices to be implemented to achieve this goal. Finally, clinicians should be provided with dedicated training to acquire the knowledge and skills needed to engage effectively with their patients: discussion of clinical cases, role-playing, consultation simulations, and shared supervision are important initiatives in this area.

### Fourth: *Benefit from the Family Caregiver Boost!*

Experts believe that family caregivers are catalysts of clinicians’ actions, not obstacles to them. Partnering with them is an important step toward ensuring the most effective patient engagement. Family caregivers are fundamental for enhancing patients’ motivation to comply with clinicians’ prescriptions and improving their engagement. Furthermore, also family caregivers need dedicated education and support in order to acquire the knowledge and skills necessary to sustain and foster the patient’s engagement: leaflets, books, multimedia platforms, learning videos, websites, seminars/workshop/conferences are today widely used to educate caregivers. However, this does not appear enough: the emotional burden of caregivers and their subjectivity in the care process need to be addressed in order to maximize the benefits of clinicians’ interventions. Motivational interviews, goal setting, problem-solving techniques, wellness plans, behavioral counseling and mindfulness interventions are recommended as important strategies to achieve these goals. Finally, social stigma, insensitivity and unawareness are common causes of patients’ and family caregivers’ burdens; these aspects make it difficult to cope actively with the disease and its management. Efforts should be made to sensitize society further about the unmet needs of patients and caregivers and about the importance of patient engagement. To achieve true engagement, patients need to be considered as persons fully integrated into their family and community. For this reason, also the society needs to be sensitized to the patient’s engagement goal.

## What Next? Joining Efforts for A Patient Engagement Cultural Change

Promoting effective patient engagement is a long and complex process that needs continuous fine-tuning between evidence from scientific research and clinical practices. The voices of patients and families in the process need also to be given greater consideration. In other words, actualization of the patient engagement imperative requires a profound cultural change in how healthcare services are designed and delivered. The ICCPE (which is going to be concluded in June 2017) will take a first small step forward in the definition of shared guidelines for patient engagement practices. We call for clinicians, healthcare professionals, policy makers, patients, families, and citizens to join their efforts in further definition of what may sustain patient engagement and what should be the golden rules to be followed. We truly believe that only a multi-disciplinary and multi-stakeholder consensus can transform patient engagement from “a fashionable phrase” to a shared guideline for practice. The ICCPE is being conducted in Italy and with Italian experts. Thus the recommendation that will emerge will be surely applicable for the Italian context. However, since the ICCPE experts discussed and shared their best practices of engagement also in the light of an extensive analysis of the scholarly international literature about patient engagement, it may be considered that the principles emerged from this work may be generalized to other healthcare system. Future work should assess the cross-cultural validity of emerging recommendations.

## Author Contributions

GG designed and managed the research process. GG and SB drafted the manuscript; GR, WR, ACB supervised the process; SB, MS, JM, MC, GG, and AT helped in designing, coordinating, and managing the process. The other authors contributed with their feedback to the final version of the manuscript. All the authors have a longstanding interest in patient engagement research and policies. GG, SB, and GR participated in the conception of this project. GG and SB performed the literature review and took primary responsibility for initial drafting of the manuscript. GR, WR, and ACB gave important supervision for drafting the final version of the manuscript.

## Conflict of Interest Statement

The lead author GG affirms that the manuscript is an honest, accurate, and transparent account of the study being reported; that no important aspects of the study have been omitted; and that any discrepancies are disclosed. The other authors declare that the research was conducted in the absence of any commercial or financial relationships that could be construed as a potential conflict of interest.
